# The performance of the EMS triage (RETTS-p) and the agreement between the field assessment and final hospital diagnosis: a prospective observational study among children < 16 years

**DOI:** 10.1186/s12887-019-1857-0

**Published:** 2019-12-16

**Authors:** Carl Magnusson, Johan Herlitz, Thomas Karlsson, Maria Jiménez-Herrera, Christer Axelsson

**Affiliations:** 1 Department of Molecular and Clinical Medicine, Institute of Medicine, Sahlgrenska Academy, University of Gothenburg, Gothenburg, Sweden; 2 Pre Hospen-Centre for Prehospital Research, Faculty of Caring Science, Work Life and Social Welfare, University of Borås, Borås, Sweden; 3 Health Metrics Unit, Sahlgrenska Academy, University of Gothenburg, Gothenburg, Sweden; 4 Department of Nursing, Rovira i Virgili University, Tarragona, Spain

**Keywords:** Triage, Accuracy, Emergency medical services, Patient assessment, Paediatric emergency care

## Abstract

**Background:**

The rapid triage and treatment system for paediatrics (RETTS-p) has been used by the emergency medical services (EMS) in the west of Sweden since 2014. The performance of the RETTS-p in the pre-hospital setting and the agreement between the EMS nurse’s field assessment and the hospital diagnosis is unknown. The aim of this study was to evaluate the performance of the RETTS-p in the EMS and the agreement between the EMS field assessment and the hospital diagnosis.

**Methods:**

A prospective observational study was conducted among 454 patients < 16 years of age who were assessed and transported to the PED. Two instruments were used for comparison: 1) Classification of an emergent patient according to predefined criteria as compared to the RETTS-p and 2) Agreement between the EMS nurse’s field assessment and the hospital diagnosis.

**Results:**

Among all children, 11% were identified as having vital signs associated with an increased risk of death and 7% were diagnosed in hospital with a potentially life-threatening condition. Of the children triaged with RETTS-p (85.9%), 149 of 390 children (38.2%) were triaged to RETTS-p red or orange (life-threatening, potentially life-threatening), of which 40 (26.8%) children were classified as emergent. The hospitalised children were triaged with the highest frequency to level yellow (can wait; 41.5%). In children with RETTS-p red or orange, the sensitivity for a defined emergent patient was 66.7%, with a corresponding specificity of 67.0%. The EMS field assessment was in agreement with the final hospital diagnosis in 80% of the cases.

**Conclusions:**

The RETTS-p sensitivity in this study is considered moderate. Two thirds of the children triaged to life threatening or potentially life threatening were later identified as non-emergent. Of those, one in six was discharged from the PED without any intervention. Further, one third of the children were under triaged, the majority were found in the yellow triage level (can wait). The highest proportion of hospitalised patients was found in the yellow triage level. Our result is in agreement with previous studies using other triage instruments. A computerised decision support system might help the EMS triage to increase sensitivity and specificity.

## Background

Increased demand for paediatric emergency department (PED) resources has led to the necessity to utilise triage systems in the PED when the demand exceeds the resources that are available. The requirements relating to a triage system are that it has to be safe, reproducible and efficient [1]. Furthermore, the triage system should demonstrate validity and reliability and also be of relevance for the assessment of the patient. The aim of a triage system is to rapidly aid in the assessment of the patient and categorise patients based on the severity of the condition [2]. Patients with critical conditions are prioritised to reduce the waiting time for a physician evaluation [3]. Triage systems are constructed based on expert opinion and, as such, there is no gold standard for what constitutes a “true” emergency [4, 5], but five-level priority scales have shown better accuracy compared with three-level scales in order to identify the most critically ill patients [2, 6]. Internationally, the dominant triage systems are the emergency severity index (ESI), the Manchester triage system (MTS), the Australasian triage scale (ATS), the South African triage scale (SATS) and the paediatric Canadian triage scale (pCTAS). Previous studies have reported moderate to good inter-rater reliability and internal validity [5, 7]. The ESI has achieved a better performance in predicting hospital admission compared with the MTS, ATS and pCTAS [8]. Furthermore, the ESI, MTS and pCTAS have also achieved a higher performance when studied in the country/region where they were developed compared with being exported and implemented in other countries [9]. In Sweden, the majority of the PEDs at university hospitals use the rapid triage and treatment system for paediatrics (RETTS-p), a five-level triage system which was developed in Sweden and initially implemented in 2010. In 2014, the RETTS-p was introduced in the emergency medical services (EMS) in the western part of Sweden to start the triage process and assess the level of severity at an earlier stage. There is a lack of knowledge relating to the accuracy of pre-hospital triage and the performance of the RETTS-p in the EMS. The aim of this study was therefore to evaluate the accuracy of the pre-hospital RETTS-p and the agreement between the EMS assessment and the final hospital diagnosis among patients < 16 years of age allocated to the PED by the EMS nurse.

## Methods

### Design

This study is an observational prospective study with a retrospective analysis of paediatric patients assessed by an EMS nurse and transported to the PED. Prospectively, we informed the EMS staff about the study and educated the staff at several workplace meetings at all nine of the ambulance stations in the EMS organisation. The education was made up of a repetition of the triage process with the RETTS-p triage protocol and also repetition of patient assessment according to local guidelines. This was further emphasised in official weekly newsletters sent out to all the EMS staff before and during the study. The purpose was to minimise bias and to achieve a uniform patient assessment and also to increase the data quality of the prehospital patient records.

### Study population

Patients were eligible to be included in the study if they had had contact with dispatch and had been assessed as being in need of prehospital emergency care. The first thousand EMS assignments a month were collected consecutively in 2016 (*n* = 12,000) as a convenient sample. All children below 16 years of age in this sample were included if the inclusion criteria were met. The inclusion criteria were: 1) < 16 years of age and thus eligible for paediatric care at the children’s hospital and 2) primary assessment by an EMS nurse at the scene and assessed at the PED. The exclusion criteria were: 1) inter-hospital transport, 2) assistance to other ambulance, 3) assignments with no patient contact. A total of 716 children were initially identified in the sample and 65 children were excluded as they did not fulfil the inclusion criteria. Informed consent was not required for this EMS record registry based study. Informed consent is mostly not recommended by Ethical Review Boards in Sweden in this type of study for the following reasons. 1) Individual patients could never be identified in the analysis, since their identification number was translated to a code. Their integrity thus remained unaffected. 2) Some of the most severe cases could never be contacted in retrospect as they had either died or were in a very poor clinical condition. Furthermore logistical reasons and difficulties to understand our language would prevent communication with a number of patients. Thus, a requirement of informed consent would increase the risk of selection bias, thereby hampering the reliability of the data. 3) Approaching patients and/or relatives about these types of issue may create more anxiety than satisfaction and may therefore be regarded as unethical.

### Settings

The study was conducted in a single-site urban setting in the western part of Sweden. The EMS organisation covers 900 km^2^, with a population of 660,000 inhabitants. The EMS carries out more than 80,000 assignments every year, of which 58,575 assignments are identified as primary assessments and approximately 3100 are children < 16 years of age.

All health care in Sweden is tax funded and free to residents of Sweden, regardless of the type of disease. In Sweden, the twenty-one county councils are responsible for providing health care for the residents within the county, including the EMS. The EMS can be organised within the body of a university hospital, county or contracted to a private company. In the study organisation, the EMS is organised under the university hospital. The EMS has 22 units, of which 18 are ambulances all with advanced life-saving (ALS) capacity and four special units; two single responders manned by one specialised trained registered nurse, one emergency physician-manned unit and a scene-command unit. Within the urban area, there is a designated children’s hospital with full capacity to handle all kinds of paediatric ailment, resulting in short transportation times from the scene to the PED. In total, the EMS carries out approximately 5.4% runs annually with children < 16 years of age. The retrospective patient data were collected from the EMS organisation patient records and from the hospital medical records for the specific in-hospital care event. Each record was reviewed manually and unidentified data such as vital signs, triage level, EMS nurse assessment and ICD code were then entered into a registry of which statistical analyses were conducted in a aggregated form.

### RETTS-p triage system

The RETTS-p triage system is similar to the MTS triage system in its organisational structure, with flowcharts for each presentation. In order to identify patients at risk of deteriorating, the RETTS-p has included vital signs (VS) in each flowchart. The RETTS-p was developed and is maintained and licensed by a Swedish company (Predicare AB). The RETTS-p consists of two components, VS and emergency symptoms and signs (ESS). VS (SaO2, respiratory rate, pulse rate, temperature and level of consciousness) are recorded for each patient, together with an ESS stating the level of severity. The highest triage level of the VS or ESS constitutes the final triage level. The patient is triaged to a triage level colour ranging from red, defined as life threatening, orange potentially life threatening, yellow, green and blue. Red and orange are considered to be in need of a direct medical evaluation by a physician to what is defined as an acute process. The triage levels of yellow and green can wait without jeopardising the individual medical risk, but yellow is regarded as a higher level due to deviating VS or signs and symptoms (ESS) that should be evaluated by a physician before green-triaged patients. The blue level is the lowest triage colour and alternatives other than the PED may be more appropriate. At the time of this study, only triage levels green to red were used in the EMS nurse triage.

### Prehospital management of the paediatric patient

When a patient dials the Swedish emergency number (112), a dispatcher assesses the patient according to a medical index and assigns the patient a priority of 1 to 4. Priority 1 is life threatening and one or two ALS units respond to the patient with blue lights and sirens. Priority 2 is considered urgent and a unit should respond within 30 min. Priority 3 can wait without medical risk. Priority 4 is assigned when there is a need for transport only and it is carried out by a non-emergency patient transport manned by one emergency medical technician. For priority 1–3, it is mandatory according to legislation in Sweden that a registered nurse conducts the assessment at the scene and is responsible for the administration of drugs. All ambulances in Sweden are therefore manned by a registered nurse, often with a postgraduate education in prehospital emergency care. Ambulance crew set-ups can be two registered nurses or one nurse and one EMT. The EMS nurse has been given the responsibility independently to decide upon the level of care which includes 1. Transport to hospital, 2. Arrange an appointment at a primary care centre and 3. Treat and release and/or give advice on self-care. To aid in the assessment, the EMS nurse uses the triage system (RETTS-p) to help discriminate between degrees of severity and a telephone consultation with a paediatrician at the PED, especially in cases where the patient is non-conveyed, is encouraged. The EMS nurse’s on-the-scene triage level becomes the triage level at the PED. To increase patient safety, the handover to the PED nurse always takes place together with the patient/ significant other.

### Evaluation of the RETTS-p triage accuracy

Defining a true emergency patient is problematic and, as a result, no gold standard exists [10]. Predominantly, two options are available when aiming to test the internal validity of triage systems: 1. by proxy variables, for example, interventions performed and/or the need for hospitalisation and 2. physiological factors such as vital signs or blood samples. We have used a mixture of these in this study and a similar approach has been used when evaluating the MTS [10–12]. An “emergent” patient is defined as having abnormal VS according to the definition or a potentially life-threatening condition according to the final diagnosis. Reference VS that are compared against the RETTS-p VS are based on the paediatric increased risk of mortality acute physiology score III (PRISM III-APS) [13]. An increased risk of mortality in the PRISM-APS has been regarded as a qualifier for an “emergent” patient. Furthermore, SaO2 is part of the RETTS-p VS, but, in the PRISM III-APS, PaO2 is reported. SaO2 has been converted to PaO2 using a formula presented by Severinghaus, defining SaO2 of < 91% as the outside reference with an increased risk of mortality [14] Additional file 1. Potentially life-threatening conditions are defined according to conditions presented in previous studies [10, 11]. Examples of these conditions are sepsis, meningitis, high-energy trauma, substantial blood loss and intoxication Additional file 2. We have used a sequential step comparison of 1. qualification against VS and 2. comparison against defined potentially life-threatening conditions. This is an approximation of criterion validation (true emergency) and the RETTS-p triage levels of red or orange fulfilling the criteria are regarded as a true positive.

### The agreement of the EMS field assessment at the scene compared with hospital diagnosis

The EMS assessment, in addition to the triage level, has been collected from EMS records, where the EMS nurse registers the assessed condition and/or symptoms. In order to compare the EMS field assessment with the final physician diagnosis in hospital, a diagnosis classification instrument has been used. This instrument categorises life-threatening diagnoses and non-life-threatening diagnoses and has previously been used in another study [15]. It has also been further explained in a study protocol [16]. The instrument comprises five categories (A-E) and is described in Table 4. In cases where it was difficult to determine the category, the case was discussed in a group to reach consensus about the classification.

### Statistical analysis

The results in this study are presented as numbers and percentages or as the median with 25th and 75th percentiles. Missing data have been denoted in each table and, in Table 3, only patients recorded with a final triage colour in the ambulance were included. In Table 1, group comparisons were performed using Fisher’s exact test for categorical variables and the Mann-Whitney U test for continuous variables. To calculate sensitivity, specificity, positive and negative predicted values and likelihood ratios, data from Table 3 were used, where RETTS-p levels red and orange were regarded as a positive test and an emergent patient was defined as having either life-threatening VS or a potentially life-threatening condition. Under-triage and over-triage was defined as 1-Sensitivity (proportion of yellow/green triaged among all emergent patients) and 1-Specificity (proportion of red/orange triaged among all non-emergent patients) respectively. All tests are two-sided and *p*-values below 0.01 are considered significant due to the number of tests in Table 1. SPSS version 22 was used to perform data processing and statistical analysis.

**Table 1:** Patient characteristics of EMS RETTS-p triaged patients vs. No triage colour

	EMS triage colour	EMS No triage colour	Total	*P*^*1*^
	*n* = 390	*n* = 64	*n* = 454	
Age – months (Q1,Q3)				
Median	59 (20, 141)	23 (14, 50)	50 (18, 136)	< 0.001
Sex – *n* (%)				0.006
Male	213 (54.6)	47 (73.4)	260 (57.3)	
Female	177 (45.4)	17 (26.6)	194 (42.7)	
PED admission time of day – n (%)				0.468
08:00–16:00	137 (35.1)	20 (31.3)	157 (34.6)	
16:00–24:00	187 (47.9)	36 (56.3)	223 (49.1)	
24:00–08:00	66 (16.9)	8 (12.5)	74 (16.3)	
PED flow time – hh:mm (25th, 75th percentile)^2^ (4,2)^3^				
Median	2:45 (1:46, 4:11)	2:18 (1:38, 3:39)	2:41 (1:45, 4:02)	0.086
Time to physician – hh:mm (25th, 75th percentile)^4^ (28,6)^3^				
Median	1:33 (0:53, 2:38)	1:11 (0:43, 1:43)	1:28 (0:51, 2:31)	0.016
Medical history – n (%)^5^				
No prior medical history	254 (64.9)	43 (67.2)	297 (65.4)	0.779
Congenital disability	25 (6.4)	5 (7.8)	30 (6.6)	0.595
Asthma, common cold with asthma	23 (5.9)	4 (6.3)	27 (5.9)	0.782
Febrile non-epileptic convulsions, absences	23 (5.9)	1 (1.6)	24 (5.3)	0.227
Allergies	18 (4.6)	0 (0.0)	18 (4.0)	0.090
PED speciality				0.582
Medical	265 (67.9)	45 (70.3)	310 (68.3)	
Surgical	83 (21.3)	15 (23.4)	98 (21.6)	
Orthopaedics	42 (10.8)	4 (6.3)	46 (10.1)	
Management PED – n (%)				0.885
Discharged with no intervention	122 (31.3)	22 (34.4)	144 (31.7)	
Discharged with intervention by physician	162 (41.5)	25 (39.1)	187 (41.2)	
Admitted to inpatient care	106 (27.2)	17 (26.6)	123 (27.1)	
Days of inpatient care – n (Q1, Q3)^6^				
Median	2 (1,3)	4 (2,13)	2 (1,4)	0.019
Patients with emergent condition – n (%)^7^				
Vital signs with increased mortality risk > 2.5 times (1, 2)^3^	42 (10.8)	8 (12.5)	50 (11.0)	0.688
Potentially life-threatening diagnosis	26 (6.7)	5 (7.8)	31 (6.8)	0.788
Mortality 30 days – n (%)	2 (0.7)	3 (4.7)	5 (1.1)	0.022

^1^*P*-value calculated on group’s triage colour and no colour at 0.01 level

^2^Median time from *PED* admission to discharge or admission to inpatient care

^3^Missing patients in EMS triage colour group and *EMS* no triage colour respectively

^4^Elapsed median time from patient call to physician evaluation

^5^The five most common medical histories, a patient can have more than one condition

^6^Median days of inpatient care among those patients who were admitted

^7^Four patients belong to both vital sign and diagnosis group

## Results

A total of 651 children < 16 years of age were identified in the population. Of these, 197 patients were treated at the scene or referred to primary care. A total of 454 patients were assessed and transported to the PED. From now on, the results will only deal with the patients transported to the PED.

### Patients assessed to a triage colour according to RETTS-p vs. not assessed to a triage colour

Of the 454 assessed patients, 390 (85.9%) patients received a triage colour according to RETTS-p from the EMS nurse. The group associated with a triage colour was significantly older. The majority of the patients in both groups were males, had no previous medical history, arrived at the PED between 4 pm and 12 pm, visited the PED for a medical ailment and were discharged from the PED with an intervention by a physician such as X-ray, ultrasound, suturing, blood sample, medical treatment or prescription. Of all the children, 11% were identified with VS associated with an increased risk of death and 7% were diagnosed with a potentially life-threatening condition. A total of 31.7% of all children were discharged from the PED without any intervention (Table 1).

### Accuracy of the RETTS-p

A total of 390 patients transported to the PED were assessed with a triage colour according to RETTS-p by the EMS nurse. Of them, 149 patients (38.2%) were triaged to a level red (life threatening) or orange (potentially life threatening) condition, according to RETTS-p guidelines. Of them, 55% of the red- and 31% of the orange- triaged patients were hospitalised (Table 2). According to the definition of an emergent patient, based on the classification of VS and a potentially life-threatening condition, only 40 patients (26.8, 95% CI [22.5,31.7]) at the red and orange triage level qualified as emergent patients. We found that the majority of the patients admitted to in-patient care were not classified as emergent patients according to VS or a potentially life-threatening condition (Table 3). Eight patients with life-threatening VS according to the definition had more than one VS outside the reference interval. An abnormal heart rate (*n* = 24) and an abnormal temperature (*n* = 12) were the most frequent presentations among the patients identified with VS according to the emergent patient category. If triaged to yellow or green, the predictive value of not being life threatening or potentially life threatening was 91.7, 95% CI [88.5,94.1]. The RETTS-p triage levels of red and orange had a sensitivity of 66.7% for detecting an emergent patient, 95% CI [53.3,78.3], i.e. an under-triage of 33.3%. The corresponding specificity was 67.0, 95% CI [61.6,72.0], i.e. an over-triage of 33.0%. The positive likelihood ratio was 2.02, 95% CI [1.59, 2.56], and the negative likelihood ratio was 0.47, 95% CI [0.33, 0.67].

**Table 2:** EMS triage level according to RETTS-p and hospitalisation

	Red	Orange	Yellow	Green	Total
Admitted to inpatient care^1^ (17)^2^	22	34	44	6	106
PED discharge with intervention (25)	13	49	73	27	162
PED discharge with no intervention (22)	5	26	63	28	122
	40	109	180	61	390

^1^Categories denoted in number of patients and one patient per category

^2^Missing triage colour per category

**Table 3:** Pre-hospital triage level according to RETTS-P in comparison with emergency definition

	Red	Orange	Yellow	Green	Total
Life-threatening vital signs^1^ (8)^2^	13	11	16	2	42
Potential life-threatening diagnosis (2)	4	12	1	1	18
Admitted to inpatient care (10)	9	25	39	6	79
PED discharge with intervention (22)	10	40	70	25	145
PED discharge with no intervention (22)	4	21	53	28	106
	40	109	179	62	390

^1^Categories denoted in number of patients and one patient per category

^2^Missing triage colour per category

### Agreement between EMS field assessment and hospital diagnosis

A total of 412 of 454 patients transported to the PED received a hospital diagnosis (ICD-10-SE). The remainder were treated by a nurse practitioner in the PED or the patient left the PED before being examined by a physician. Thirteen patients (3.2%) were classified with a life-threatening specified hospital diagnosis and 320 patients (77.6%) were classified with a specified hospital diagnosis but not life threatening. Another 79 patients had a final diagnosis described as a symptom (for example, dyspnoea) or a diffuse assessment such as observation or evaluation of a non-defined incident. The EMS nurse’s field assessment was in agreement with the hospital diagnosis in 329 (80%) of the cases. Among the remaining 83 cases, the field assessment was not in agreement with the hospital diagnosis or the symptoms reported by the EMS nurse were atypical or uncommon for the hospital diagnosis. In 33 patients (8%), the EMS field assessment diverged from the hospital diagnosis. One of these patients had a life-threatening hospital diagnosis with a fatal outcome in the PED (Table 4).

**Table 4:** Agreement between the EMS field assessment and the final diagnosis

	EMS field assessment (*n* = 412)^1^
A. A defined final diagnosis classified as life threatening	*n* = 13
1. The field diagnosis is in agreement with the final diagnosis	5 (38.5)
2. The field diagnosis is not in agreement with the final diagnosis	1 (7.7)
3. Typical symptoms related to the final diagnosis	7 (53.8)
4. Atypical symptoms related to the final diagnosis	0
5. More unusual symptoms related to the final diagnosis	0
6. Field assessment as a non-specified organ system	0
B. A defined final diagnosis not classified as life threatening	*n* = 320
1. The field diagnosis is in agreement with the final diagnosis	142 (44.4)
2. The field diagnosis is not in agreement with the final diagnosis	25 (7.8)
3. Typical symptoms related to the final diagnosis	120 (37.5)
4. Atypical symptoms related to the final diagnosis	13 (4.1)
5. More unusual symptoms related to the final diagnosis	14 (4.4)
6. Field assessment as a non-specified organ system	6 (1.9)
C. The final diagnosis is expressed as a symptom	*n* = 70
1. The field diagnosis is in agreement with the final symptom	2 (2.9)
2. The field diagnosis is not in agreement with the final symptom	5 (7.1)
3. Field symptom and the final symptom are in agreement	49 (70.0)
4. Field symptom and the final symptom are not in agreement	7 (10.0)
5. Field assessment as a non-specified organ system	7 (10.0)
D. The final diagnosis is described as a non-specific assessement	*n* = 9
1. The field diagnosis is in agreement with the symptom	2 (22.2)
2. The field diagnosis is not in agreement with the symptom	2 (22.2)
3. Field symptom is in agreement with the final assessment	2 (22.2)
4. Field symptom and final assessment are not in agreement	3 (33.3)
5. Field assessment presented as a non-specified organ system	0

^1^ Total number of final in-hospital diagnoses in 454 patients

## Discussion

In the present study, we evaluated the accuracy of the RETTS-p triage system in an EMS setting and the EMS nurse’s assessment compared with hospital diagnosis. To our knowledge, this is the first study to evaluate a PED triage system in the prehospital setting and the EMS nurse’s assessment in comparison with the physician’s assessment and outcome in hospital. The findings in this study showed a sensitivity, when red- or orange-triaged patients were later identified as emergent cases, of 68.2% and a specificity of 67.0%. The EMS nurse’s field assessment was in agreement with the physician’s hospital diagnosis in 80% of cases.

The triage sensitivity in this study is consistent with other studies of the MTS in the PED, with a reported sensitivity of 58–74% [10, 11, 17–19]. This has previously been reported as moderate sensitivity [5]. The RETTS-p specificity of 67.0% in our study was lower than that in previous MTS studies in the PED, reporting rates of 78–87% [10, 11, 17–19], but higher compared with a study from Norway, a Scandinavian country similar to Sweden with a modified paediatric SATS triage in the PED, which reported a sensitivity of 74% and a specificity of 48% [20].

Furthermore, we found an over-triage of 33.0% and an under-triage of 33.3%. Other studies of paediatric triage systems in the ED have reported an over-triage of 16–54% and an under-triage of 2–28.7% [10–12, 17, 18, 21–23]. In this study, the majority of the patients found as under-triaged were triaged to yellow level, were one year of age or below with fever or pulse rate as a deviating vital sign compared with the reference standard. Of the 20 patients who were under-triaged, three patients were hospitalised, five patients were treated in the PED and a total of 12 patients were administered drugs by the EMS nurse. The majority of the EMS under-triaged children were later diagnosed with infectious diseases and febrile seizures with a viral origin.

However, the aim of a triage system is not to identify a certain diagnosis but to identify patients at risk. Children may run an increased risk of under-triage since they have more unspecific complaints and deviations in vital signs occurring later in the process [24]. It may therefore be useful to conduct a triage at the scene combining both ESS and VP. Even though a built-in over-triage is needed, over-triaged patients consume resources. In a simulation study of mass casualty trauma care, over-triaging patients increased the overall mortality when the number of non-urgent patients increased [25]. An incorrect triage level can also be explained by misclassification. However, previous studies of the RETTS-p have reported good to very good agreement between nurses in the PED triaging children [26, 27]. On the other hand, using memory and written manual guidelines during transport may further complicate the EMS nurse triage process.

In this study, the under- and over-triaged cases were found with an even distribution over the year, which suggests that increasing triage knowledge is dependent on patient frequency, which was similar across the year. On the other hand, compared with the above-mentioned PED triage studies with high exposure of triage cases, the findings are similar. In the latest version (2019) of the RETTS-p, changes have been made to reduce under-triage, specifically for patients presenting with fever above 38.5 C (VS) and current or past symptoms of chills (ESS). They should now be triaged to orange level (potentially life threatening). In the future, this may lead to a decrease in under-triaged children but also an increase in over-triage. Designing a triage system with low under-triage, while, at the same time, not increasing over-triage, is essential and problematic. Acceptable rates of over- and under-triage have not been agreed upon. However, for trauma patients, the American College of Surgeons states an under-triage of below 5 % and 25–35% over-triage, but as much as 50% has also been mentioned [28].

Regarding admission to in-patient care, the highest frequency of patients in all categories admitted to in-patient care were triaged to yellow level. Conditions such as seizures, infections, commotio (concussion) and fractures which required surgery were found at this triage level. This may indicate a weak association between the triage level and the severity of the condition when classified according to the need for hospitalisation. However, the hospital admission rate per triage level increases with a higher urgency level from 9.8% at green level to 55% at the red triage level. The association between triage level and the need for hospitalisation has also been addressed in other studies of triage in the PED. The ESI has shown an association of 84–100% predicting hospital admission at the highest triage levels [7, 23, 29]. The proportion of patients at the red triage level in this study is relatively high (9.8%) compared with studies of the ESI, pCTAS and the MTS (0.1–2.9%) [7, 11, 29, 30]. This suggests that greater over-triage is built into the RETTS-p system compared with other triage studies in the PED.

### EMS field assessment in agreement with hospital diagnosis

Of the total number of children that were transported to the PED in this study, 13 (3.2%) were given a specified hospital diagnosis identified as life threatening, such as toxic shock syndrome, intracerebral haemorrhage or anaphylaxis. The prevalence of critical conditions in the PED has been reported in studies, with rates of 0.28–4.2%, depending on the region, patient cohort and the definition of a critically ill child [31–34]. The fact that this study only involved the patients initially in contact with the EMS may have increased the prevalence, albeit low overall. In the majority of patients, the field assessment was in agreement with the final hospital diagnosis, with only one patient classified as having a life-threatening final diagnosis which was not in agreement with the field assessment. However, the diagnosis for another 32 patients of those classified with a non-life-threatening final diagnosis was not in agreement with the EMS field assessment. Paramedics’ ability to predict hospital admission and the aetiology of the condition in adults has been shown to be moderate in several studies [35–37]. In one study, 19% of the paediatric transports with lights and sirens were later identified as unnecessary [38]. In a study of adults from Ireland, advanced paramedics showed agreement in 70% of the cases when the field diagnosis and the ED physicians’ working diagnosis were compared. Areas of less agreement included patients with no abnormalities [39]. These results suggest that it is particularly difficult to exclude a life-threatening condition, as further tests and examinations are required after arrival at hospital. Overall limited exposure and lack of training may be one reason for a less appropriate field assessment, which has been previously reported [40, 41]. Furthermore, environmental characteristics also play a role in the assessment, where the EMS is confronted by various challenges, such as worried parents, language barriers with no support in interpretation on the scene or no observable abnormality.

### Children not assessed to a triage colour according to RETTS-p

In this study, 14.1% of the patients were not triaged to a colour, which can be explained in several ways. Firstly, short transportation times may have influenced the triage before arrival at the PED. Secondly, patients were significantly younger in the non-triage group, indicating difficulties either in the EMS nurse’s competence assessing infants according to the triage system or in obtaining VS in these patients. A lack of VS in paediatric patients in the pre-hospital setting has also been previously reported, with an increasing rate of fewer recorded VS in younger children [42, 43]. This is worrying, as the youngest children run a higher risk of serious bacterial infections [10, 44]. Thirdly, some of these patients were transported directly to the emergency room and the EMS nurse may have been occupied with patient interventions before arrival at the PED. As a result, the patient was assessed to the highest triage level, even though this was not recorded. However, the number of patients not triaged to a colour in this study appears to be similar to studies of other triage systems, such as the MTS in the PED, which reported a missing triage level in 5–16% of the patients [10–12, 45]. On average, the EMS nurses in the study organisation assess approximately 50 paediatric patients every year in an unselected patient population. This may indicate that the lack of exposure has an impact on triage knowledge and assessment. On the other hand, the use of a triage system by the EMS clinician at the scene in every case may help to identify patients at risk in a systematic approach when assessing patient severity. A triage system with both VS and ESS may contribute to increased patient safety when utilised in a setting and used by nurses with limited exposure to paediatric conditions, especially since the ESS part of the system acts as a checklist based on severity for each condition within the triage system. However, adherence to guidelines and protocols has been reported with large variability and suboptimal adherence for certain patient groups in the EMS (7.8–95%) [46]. One survey of EMS nurses reported adherence to guidelines of 84% and one factor explaining the variation in adherence was the characteristics of the guidelines [47]. Using manual triage systems, where the EMS nurse has to recall from memory when documenting the triage process, can be problematic. A study of the adult CTAS revealed improved performance when implementing a computerised triage system compared with traditional manual triage [48]. Introducing an electronic version of the RETTS-p adapted for pre-hospital use and incorporated in a decision support system, in combination with regular training on paediatric assessment, may increase adherence to and confidence in the system. Furthermore, the introduction of dedicated units for paediatric cases with staff rotation into the PED would increase exposure and competence in the at-the-scene assessments.

#### Strengths and limitations

The data in this study were collected consecutively from a relatively large group from all the eligible patients in contact with the EMS where the data were reported prospectively. Another strength is that the EMS nurse was not aware of the situation in the PED regarding flow time and triage levels. The PED nurse triaging a patient is constantly aware of this. It is defined as triage drift, where the PED nurse may overrule the triage system for a specific patient for logistical reasons. There are good reasons to discuss several limitations. This is a relatively small study. Despite being collected from a relatively large sample, the frequency of children in the EMS is limited. Furthermore, patient assessment is a dynamic process and triage should be seen as a sequential process over time. For this reason, any deterioration in a condition or improvement due to an intervention by the EMS nurse may have an impact on the patient’s condition and triage at a later stage of the chain of care. However, in the study, short transportation times and the fact that the final triage colour and the last recorded VS have been taken into consideration have minimised the risk of this. Furthermore, the potential for patient records being recorded with inaccurate levels and VS must be considered. This study was conducted at a single site and it may therefore be problematic to extrapolate the results of this study. However, information about the use of a triage system in a pre-hospital context could be generalised. The definition of an emergent patient is constructed and should be interpreted as such. Using the PRISM III-APS instead of PRISM III, the mortality risk reference interval for VS is increased, detecting even smaller changes. This may have included more children in the emergent VS category. On the other hand, including these children who may be at risk of death according to the PRISM III-APS appears valid.

## Conclusions

When used by the EMS, the RETTS-p performed at the same level as other triage systems in the PED. The sensitivity of the RETTS-p in this study is considered to be moderate. The majority of under-triaged patients were found at the yellow triage level, which may be a threat to patient safety later in the chain of care. Even though it may be better to err on the safe side, it seems that the relatively large number of non-emergent red and orange patients could have yielded less under-triage than reported in this study. This indicates that the triage of certain subgroups of patients needs to be further addressed. A computerised decision support system with an adapted pre-hospital triage version could be proposed, aiming at increasing sensitivity and specificity. More studies are needed to further study the use of hospital triage systems in the EMS.

## Supplementary information

**Additional file 1.** The paediatric risk of mortality III – acute physiology score (PRISM III-APS): Life-threatening vital signs for paediatrics according to the PRISM III-APS.

**Additional file 2.** Paediatric life-threatening conditions: a list of conditions considered to be time-sensitive.

## Data Availability

The datasets analysed during the current study are available from the corresponding author on reasonable request.

## Abbreviations

ALS: Advanced life support
ATS: Australasian triage scale
EMS: Emergency medical services
ESI: Emergency severity index
ESS: Emergency signs and symptoms
ICD-10: International statistical classification of diseases and related health problems – tenth revision
MTS: Manchester triage system
Ped CTAS: Pediatric Canadian triage and acuity scale
PED: Paediatric emergency department
PRISM III-APS: Paediatric increased risk of mortality III – acute physiology score
RETTS-p: Rapid emergency triage and treatment system for paediatrics
VS: Vital signs
