# Supplementary material for: The performance of the EMS triage (RETTS-p) and the agreement between the field assessment and final hospital diagnosis: a prospective observational study among children < 16 years
Source: BMC Pediatr. 2019 Dec 16;19:500. doi: 10.1186/s12887-019-1857-0 (PMC6912993; doi:10.1186/s12887-019-1857-0)
Supplement: Supplementary file 1 — Additional file 1. The paediatric risk of mortality III – acute physiology score (PRISM III-APS): Life-threatening vital signs for paediatrics according to the PRISM III-APS. [file 12887_2019_1857_MOESM1_ESM.docx]

Additional file 1

Life-threatening vital signs for paediatrics according to PRISM III-APS

Age categories:

Neonates (N) 0 - < 1 month

Infants (I) 1- <12 months

Children (C) 12- <144 months

Adolescent (A) > 144 months

The vital sign cut off for each age category and the mortality risk ratio in parenthesis.

1. Saturation all ages: PaO2 <61mmHg (4.196)

2. Respiratory rate/min: N > 100, I > 100, C > 80, A > 60 (2.501)

3. Heart rate/min: N < 75 >194, I <75 >194, C < 55 > 164, A < 55 >134 (3.493, 2.915)

4. Level of consciousness all ages: GCS < 8 (19.114)

5. Body temperature all ages: <33 >40 (30.940, 5.805)

Pollack MM, Patel KM, Ruttimann UE. The Pediatric Risk of Mortality III--Acute Physiology Score (PRISM III-APS): a method of assessing physiologic instability for pediatric intensive care unit patients. J Pediatr. 1997;131(4):575-81.
