# Supplementary material for: The performance of the EMS triage (RETTS-p) and the agreement between the field assessment and final hospital diagnosis: a prospective observational study among children < 16 years
Source: BMC Pediatr. 2019 Dec 16;19:500. doi: 10.1186/s12887-019-1857-0 (PMC6912993; doi:10.1186/s12887-019-1857-0)
Supplement: Supplementary file 2 — Additional file 2. Paediatric life-threatening conditions: a list of conditions considered to be time-sensitive. [file 12887_2019_1857_MOESM2_ESM.docx]

Additional file 2

Paediatric life-threatening (potentially) conditions

- Meningitis
- Sepsis
- High-energy trauma
- Substantial blood loss
- Aortic dissection
- Signs of dehydration ≥10 %
- Near/ drowning
- Electrical trauma
- Apparently life-threatening event (alte)
- Intoxication
- Burns ≥10 %
- Inhalation trauma

Roukema J, Steyerberg EW, van Meurs A, Ruige M, van der Lei J, Moll HA. Validity of the Manchester Triage System in paediatric emergency care. Emerg Med J. 2006;23(12):906-10.

van Veen M, Steyerberg EW, Ruige M, van Meurs AH, Roukema J, van der Lei J, et al. Manchester triage system in paediatric emergency care: prospective observational study. BMJ. 2008;337:a1501.
